# Knowledge and experience of physicians during the COVID-19 Pandemic: A global cross-sectional study

**DOI:** 10.1371/journal.pgph.0000639

**Published:** 2022-07-29

**Authors:** Rania Mansour, Smrithi Rallapalli, Hamreet Kaur Baidwan, Mohammad S. Razai, Linda Abou-Abbas

**Affiliations:** 1 St George’s Hospital Medical School, St George’s University of London, London, United Kingdom; 2 Population Health Research Institute, St George’s University of London, London, United Kingdom; 3 Neuroscience Research Center, Faculty of Medical Sciences Lebanese University, Hadath, Lebanon; The University of the West Indies, BARBADOS

## Abstract

Physicians are on the frontline of the COVID-19 pandemic with responsibility to manage the disease. The aim of this study is to investigate physicians’ knowledge, attitudes, perceptions and experiences, as well as preventative practices regarding the COVID-19 pandemic and COVID-19 vaccinations. Further, we explore physicians’ recommendations for future pandemics. A mixed-methods online survey was disseminated to physicians globally. The survey was distributed via social media from August 9–30, 2021. Data collected included sociodemographic characteristics, knowledge, attitudes, and practices towards COVID-19, concerns regarding vaccinations, and perspectives on policies implemented. Descriptive statistics were reported, and qualitative data were analysed using inductive thematic analysis. A total of 399 physicians from 62 countries completed the survey, with similar participation from High Income Countries and Low- or Middle-Income Countries. Most physicians (87%) revealed a good level of knowledge while only half (54%) reported adhering to adequate preventative measures. More than half of participants (56%) indicated that the policies implemented to handle COVID-19 by their public health agencies were insufficient or disorganised. While most physicians reported increased mental stress (61%) and described their experience with COVID-19 using negative terminology (63%), most physicians (87%) indicated they are willing to continue working in healthcare. Physicians globally possessed good knowledge of COVID-19 and COVID-19 vaccinations; yet improvements in ensuring compliance with preventative measures is warranted. Findings from this study have important implications. As recommended by physicians, efforts to manage pandemics should involve (1) strengthening health systems, (2) minimising adverse effects of infodemics, (3) delegating decision-making roles appropriately, and (4) acknowledging global responsibility.

## Introduction

Coronavirus disease (COVID-19), caused by SARS-CoV-2 virus, has so far claimed the lives of more than 6.2 million people as of 10 May 2022 including healthcare professionals (HCPs) [1–3]. According to reports from Amnesty International and the World Health Organization (WHO) more than 115,000 HCPs have died from COVID-19, leaving an irreplaceable gap in pandemic response worldwide [4–6]. The impact of COVID-19 on HCPs has been summarized by the WHO and consists of four concerns: (1) availability and distribution of healthcare personnel and equipment, (2) health of physicians, including the risk of burnout and mental disorders, (3) social wellbeing, such as discrimination and concern for family, and (4) working conditions, such as lack of incentives, psychological support, or vaccinations [6]. Nevertheless, HCPs continue to serve on the frontline against COVID-19, often following local, national, and international preventative and treatment guidelines on the prevention and treatment of COVID-19. Further, the response and mitigation measures to control the pandemic have been updated regularly around the world and COVID-19 vaccines have been manufactured and deployed at a rapid pace. It is therefore essential that HCPs have access to relevant updated information to protect themselves against COVID-19 and to ensure appropriate patient management.

To our knowledge, previous cross-sectional studies exploring the knowledge and experiences of HCPs towards COVID-19 were primarily conducted within the first six months of the pandemic. Given the numerous changes to the guidelines since the beginning of the pandemic, these studies may not represent the current knowledge and experience of physicians. Furthermore, the majority of these studies were conducted within one country [7–22]. Results from a systematic review in November 2020 exploring these national studies revealed that HCPs possessed adequate knowledge of the disease and generally had positive attitudes towards the pandemic [13]. However, an updated systematic review in May 2021 demonstrated that HCPs had poor compliance to particular safety practices [23]. One global study conducted in March 2020, revealed that HCPs had poor knowledge regarding the virus’s mode of transmission and symptom onset [24]. Additionally, very few studies have been conducted on HCP’s perceptions of COVID-19 vaccines. These studies were also conducted on a national level and have shown that increased knowledge is an important predictor of vaccine hesitancy among HCPs [25, 26].

This global cross-sectional mixed-methods study investigates the knowledge, attitudes, perceptions, and practices (KAPP) of physicians towards COVID-19 disease and COVID-19 vaccines. Understanding the experiences of physicians globally can highlight gaps in policies and educational interventions that have been aimed at physicians and the public. Physicians’ reflections and their recommendations for future health emergencies are also explored. Future pandemics are considered inevitable due to the presence of high-risk factors such as overpopulation, poverty, and global warming [27–30]. The findings of this study and the recommendations of physicians from 62 countries will likely inform the development of future policies within health systems to support frontline health care providers during health emergencies.

## Methods

### Study design and data collection

A mixed-methods cross-sectional study using an online survey was conducted to obtain responses from physicians globally between the 9^th^ and 30^th^ of August 2021. The online survey was distributed via social media, particularly E-mail and WhatsApp, using a snowballing technique [31]. The invitation letter included a brief description of the study and a URL link to the survey. Physicians were identified via professional groups and academic institutions. In this study, a physician is defined as a medical doctor who practices medicine and includes surgical, non-surgical, and public health specialties. Informed consent was obtained by participants on the first page of the online questionnaire along with clear statements that participation was voluntary and uncompensated. To ensure quality control and to maximise completeness of the data, incomplete surveys and responses from non-physicians were removed from the analysis.

### Sample size calculation

The sample size was calculated using the online RAOSOFT sample size calculator [32]. The required sample size would be at least 377 participants for a global survey with an estimated population of more than 20,000 physicians (the largest estimate possible), in addition to an anticipated response of 50%, confidence level of 95%, and 5% margin of error.

### Survey instrument and scoring system

A structured questionnaire was designed on Microsoft Forms by the authors to cover important aspects of KAPP of physicians. The survey instrument was initially developed based on previous surveys [9, 11, 24]. The final questionnaire (S1 File) was modified for relevance based on the most recent information from the WHO Online Resources for COVID-19, as of July 07, 2021.

The final questionnaire was divided into eight sections: (1) *Sociodemographic characteristics*; (2) *Sources of information*; (3) *Knowledge section*: a total of 16 items were designed to measure physicians’ knowledge about the COVID-19 disease and vaccines. All items were single best answer questions. Correct options were assigned 1 point and incorrect options 0 points. The total knowledge score was a sum of scores. Based on Bloom’s cut-off point [11], overall knowledge was categorised as good if above 60% and poor if below 60%. Cronbach’s alpha coefficient for the knowledge questions was 0.936. (4) *Practice section*: five questions were used to evaluate utilisation of various preventative measures. The three answer options included “always”, “occasional”, or “never”. The latter two were assigned 0 points, and the former was assigned 1 point. The total practice score was a sum of scores. Physician’s overall practice was categorised based on Bloom’s cut-off point [11] as good if above 80%, and poor if below 80%. Cronbach’s alpha coefficient for the practice questions was 0.638. (5) *Physicians’ perspective on vaccinations*; (6) *Physicians’ perspective on policies implemented*; (7) *Physicians’ subjective attitudes* towards the pandemic; *(8) Physicians’ personal reflections* (Describe your COVID-19 experience in one word; What are your recommendations for future pandemics?).

Content validity of the final version was assessed by three experts who specialise in the field of infection control and emergency preparedness. The survey was then pilot tested in a sample of 10 physicians to check the acceptability, clarity, readability, and relevance of all items. Physicians did not report any problems in understanding the questionnaire. On average, the survey was completed within 10 minutes. The data of the pilot study was removed from the final analysis.

### Statistical analysis

Statistical analysis was carried out using the statistical software SPSS (Statistical Package for Social Sciences), version 22.0. Descriptive statistics were reported using means and standard deviations (SD) for continuous variables and frequency with percentages for categorical variables.

### Thematic analysis

Data from the two open-ended questions was summarised using an inductive thematic analysis approach [33]. Three team members independently coded a sample of the data until a consensus was reached and a coding framework was formulated. Two members independently coded the remaining data and negotiated agreements on discrepant codes. Three members reviewed the codes, sorted codes into descriptive categories based on patterns, and subsequently grouped descriptive categories to generate major themes.

The study was approved and given favourable ethics opinion by the St George’s, University of London Research Ethics Committee (SGREC) under study title “Knowledge and Perspectives of Health Care Providers on COVID-19: A Global Cross-Sectional Study” with REC Reference: 2021.0127. The overall study was guided by the STROBE (STrengthening the Reporting of OBservational studies in Epidemiology) Statement for cross-sectional studies [34].

## Results

### Baseline characteristics of study participants

**Fig 1:**
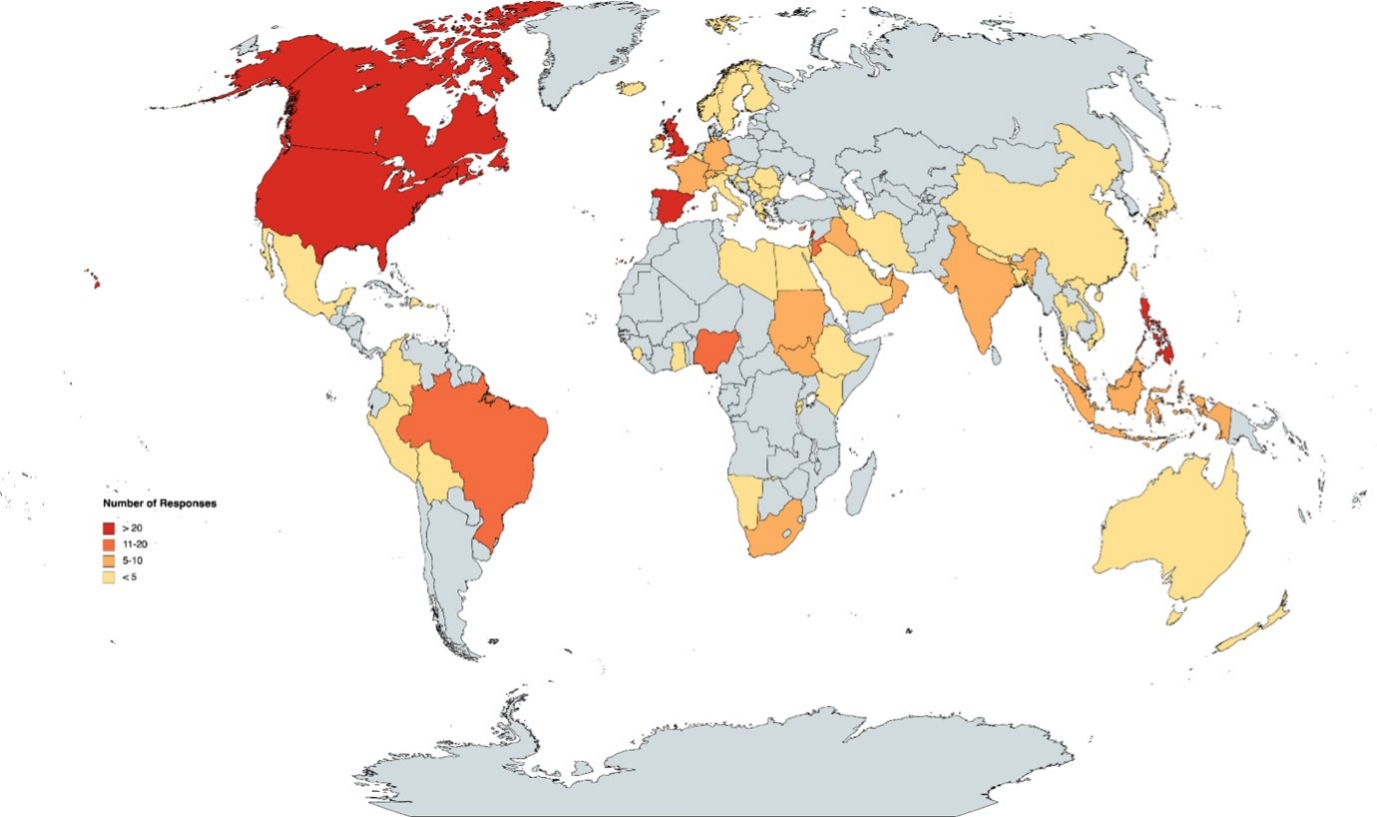
Visual representation of responses by country (n = 399). This map depicts the countries from which responses were received. More than 20 responses were received from countries shaded in red (Canada, United States of America, Spain, United Kingdom, Lebanon, Philippines). The dark orange indicate that 11 to 20 physicians responded from that country, whereas the light orange shade indicates that 5 to 10 physicians from that country responded to the survey. Less than 5 responses were received from countries shaded in yellow. No responses were received from countries in grey. Republished from https://www.mapchart.net/ under a CC BY license, with permission from Minas Giannekas, original copyright 2022.

Table 1 summarises participant characteristics. A total of 411 HCPs participated in our survey, 399 of whom were physicians, including 224 (56%) male, and 174 (44%) female. The majority were between 46–55 years old (n = 108, 27%), and practising in internal medicine (n = 80, 20%), surgery (n = 80, 20%), or general practice (n = 72, 18%). Most physicians had been practising medicine for 10 years or longer (n = 292, 73%) and most respondents identified as frontline workers (n = 268, 67%). Physicians from 62 unique countries responded to the survey, with similar participation from High Income Countries (n = 214, 54%) and Low- or Middle-Income Countries (n = 185, 46%), as identified by the World Bank [35]. Fig 1 provides a visual representation of respondents per country.

**Table 1:** Participant characteristics (N = 399).

Participant Characteristic	N (%)
**Sex**	
Female	174 (43.6)
Male	224 (56.1)
Other	1 (0.3)
**Age**	
< 25 years old	4 (1.0)
25–35	75 (18.8)
36–45	88 (22.1)
46–55	108 (27.1)
56–65	91 (22.8)
66–75	30 (7.5)
> 75 years old	3 (0.8)
**Specialty**	
Internal Medicine	80 (20.1)
Surgery	80 (20.1)
General Practice	72 (18.0)
Paediatrics	54 (13.5)
Obstetrics and Gynaecology	19 (4.8)
Emergency Medicine	15 (3.8)
Psychiatry	12 (3.0)
Radiology	12 (3.0)
Intensive Care Unit	10 (2.5)
Anaesthesiology	9 (2.3)
Family Medicine	7 (1.8)
Public Health	5 (1.3)
Other	24 (6.0)
**Frontline Worker Status**	
Yes	268 (67.2)
No	131 (32.8)
**Years of Experience**	
≥ 10 years	292 (73.2)
< 10 years	107 (26.8)
**Place of Work**	
Public Establishment	247 (61.9)
Private Establishment	152 (38.1)
**Country of Residence**	
High-Income Country	214 (53.6)
Low- or Middle-Income Country	185 (46.4)

### Sources of knowledge

Primary sources of knowledge amongst respondents were News Media and Official Government Websites (Table 2). Most physicians (51%) indicated Official Government Websites as their most-used source. The majority of respondents (43%) indicated social media as their least-used source.

**Table 2:** Sources of information (N = 399).

Response	Source of COVID-19 Information N (%)			
	News Media	Social Media	Official Govt. Website	Family member or colleague
**Least Used**	70 (17.5)	172 (43.1)	14 (3.5)	126 (31.6)
**Sometimes**	147 (36.8)	116 (29.1)	58 (14.5)	165 (41.4)
**More Used**	117 (29.3)	63 (15.8)	120 (30.1)	87 (21.8)
**Most Used**	65 (16.3)	48 (12.0)	207 (51.9)	21 (5.3)

### Physicians’ knowledge towards COVID-19 virus and vaccines

Of all physician respondents, 349 (87.5%) participants had good knowledge about COVID-19 disease and COVID-19 vaccines (Table 3). Poor knowledge was observed for questions concerning the nature of disease (52%) and treatment of disease (59.9%). Conversely, good knowledge was observed in responses regarding transmission of disease (71.5%), actions dealing with cases (72.5%), and nature of vaccines (89.5%). The mean total knowledge score was 11.07 (SD = 1.49). No differences between various physician specialties, frontline worker status, or residency in LMIC versus HIC were observed.

**Table 3:** Physician’s responses regarding knowledge of COVID-19 and vaccines (N = 399).

Knowledge Items	Physicians’ Responses (%)					
	Option 1	Option 2	Option 3	Option 4	Option 5	Option 6
**Nature of the disease**						
**K1:** What is the incubation period of COVID-19?	26.6	50.1	17.5	4.3	1.5	-
**K2:** COVID-19 origin is thought to be from:	69.7	0.0	0.3	0.0	30.1	-
**K3:** The COVID-19 variants have different clinical manifestations.	69.4	30.6	-	-	-	-
**K4:** What are the complications of COVID-19?	1.0	1.3	0.0	0.5	0.0	97.2
**Transmission of the disease**						
**K5:** COVID-19 transmission occurs through	41.1	9.0	0.3	46.4	3.3	-
**K6:** The UK and Indian variants of COVID-19 spread faster as they are more transmissible or infectious.	96.7	3.3	-	-	-	-
**Actions in Dealing with Suspected, Probable, and Confirmed Cases**						
**K7:** The use of personal protective equipment is necessary during aerosol production procedures, such as suction sputum sampling and intubation.	99.5	0.0	0.5	-	-	-
**K8:** Suspected cases of COVID-19 infection after triage should be taken into care in a negative pressure respiratory isolation room.	59.4	26.3	14.3	-	-	-
**K9:** The use of N95 masks is necessary when sampling of induced sputum from patients suspected of COVID-19 infection.	91.7	4.5	3.8	-	-	-
**Treatment of Disease**						
**K10:** Oxygen therapy should be given to all cases of severe COVID-19 with acute respiratory infection.	71.9	23.6	4.5	-	-	-
**K11:** High doses of systemic corticosteroids should be avoided in patients with confirmed or suspected COVID-19 infection and clinical manifestations of viral pneumonia.	22.6	62.7	14.8	-	-	-
**K12:** What is the treatment for COVID-19?	86.0	6.5	1.8	5.8	-	-
**Nature of Vaccines**						
**K13:** Which of the following is not a common (i.e., less than 1% chance) side effect of COVID-19 vaccines:	0.8	0.3	1.3	95.5	2.3	-
**K14:** Individuals who are immunodeficient and/or pregnant can receive the COVID-19 vaccine.	91.2	8.8	-	-	-	-
**K15:** Children below the age of 12 can receive the COVID-19 vaccine.	26.6	73.4	-	-	-	-
**K16:** The COVID-19 vaccines that are currently in development or have been approved are expected to provide at least some protection against new virus variants.	98.0	2.0	-	-	-	-

Cells highlighted in grey indicate the correct answer to each question. Please refer to the survey in S1 File for each answer option.

### Physicians’ practice towards COVID-19

Table 4 summarises preventative practices against COVID-19. Of the 399 respondents, 214 (54.1%) reported adequately adhering to preventative measures while working. The most prevalent practise among physicians was Item 3: I wash my hands with soap or rub my hands with hydro-alcoholic gel during my work shift (94.2%). Conversely, less than half of all respondents reported wearing gloves (Item 2) while working (39.6%). The mean score for overall preventative practices towards COVID-19 is 3.47 (SD = 1.18). No differences between residency in LMIC versus HIC were observed.

**Table 4:** Physicians’ responses regarding preventive practices towards coronavirus (N = 399).

Practice Items	Physicians’ Response		
	Never N (%)	Occasionally N (%)	Always N (%)
**P1:** I wear a mask while performing my job	2 (0.5)	23 (5.8)	374 (93.7)
**P2:** I wear gloves while performing my job	37 (9.3)	204 (51.1)	158 (39.6)
**P3:** I wash my hands with soap or rub my hands with hydro-alcoholic gel during my work shift	3 (0.8)	20 (5)	376 (94.2)
**P4:** I put my PPE on in the following order: 1- gown, 2- mask, 3- gloves.	79 (19.8)	84 (21.1)	236 (59.1)
**P5:** I remove my PPE in the following order: 1- gloves, 2- do hand hygiene, 3- gown, 4- mask	93 (23.3)	65 (16.3)	241 (60.4)

### Physicians’ experience towards COVID-19 vaccinations and policies

**Fig 2:**
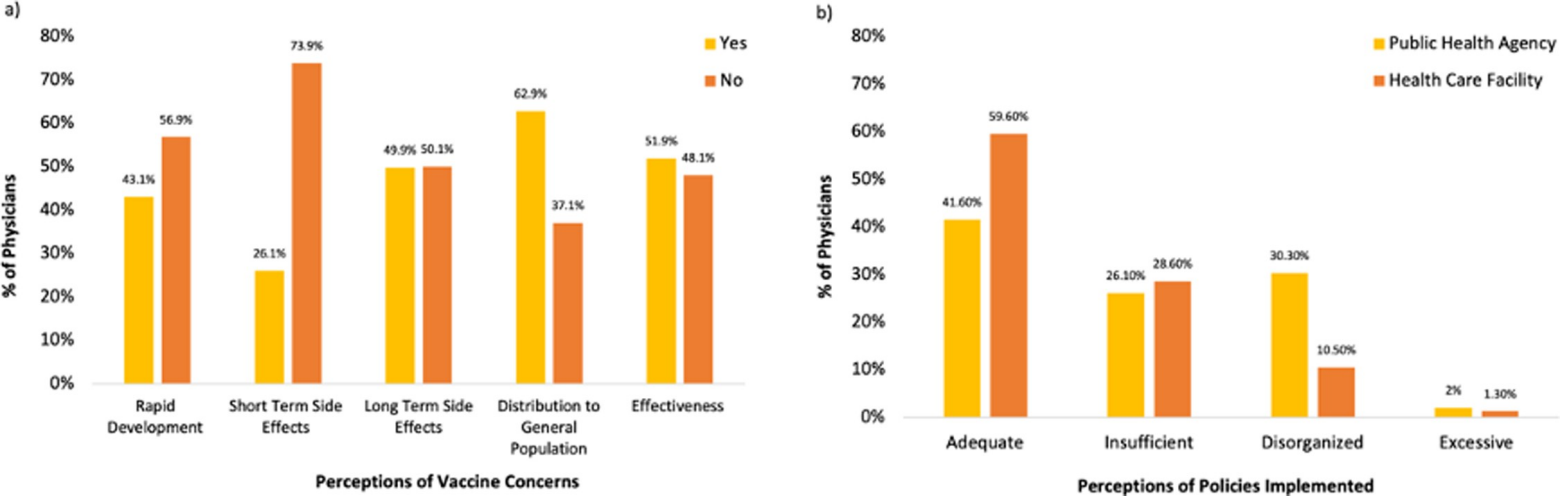
Physicians’ perceptions during the COVID-19 pandemic. Physicians’ perceptions (n = 399) regarding **(a)** various COVID-19 vaccine concerns and (b) policy actions implemented for the COVID-19 pandemic by their public health agencies and health care facilities.

Most physicians (63%) indicated being worried about distribution of vaccines to the general population and half (50%) were concerned with the long-term side effects of vaccinations (Fig 2). Table 5 summarises physicians’ perceptions towards the COVID-19 vaccines. The majority of physicians (n = 283, 71%) indicated Pfizer-BioNTech as most effective; while 195 (49%) physicians indicated that the AstraZeneca (Covishield and Vaxzevria) vaccine has the highest risk for complications, followed by the Janssen (n = 39, 9.8%) and Sputnik V (n = 38, 9.5%) vaccines. Most physicians (96%) indicated having received the COVID-19 vaccine; only 10% of physicians were/are hesitant to receive a vaccine.

**Table 5:** Summary of physicians’ perceptions towards the COVID-19 vaccines (N = 399).

Item	N (%)
**Vaccine with Highest Efficacy**	
Pfizer-BioNTech	283 (70.9)
Moderna	47 (11.8)
AstraZeneca (Covishield and Vaxzevria)	29 (7.3)
Janssen (Johnson and Johnson)	8 (2.0)
Sputnik V	7 (1.8)
Other	25 (6.3)
**Vaccine with Highest Potential for Complications**	
AstraZeneca (Covishield and Vaxzevria)	196 (49.1)
Janssen (Johnson and Johnson)	39 (9.8)
Sputnik V	38 (9.5)
Sinopharm	23 (5.8)
Sinovac Biotech	23 (5.8)
Pfizer	20 (5.0)
Moderna	9 (2.3)
Other	51 (12.8)
**Vaccination Status**	
Yes	381 (95.5)
No	13 (3.3)
Do not want to answer	5 (1.3)
**Vaccine Hesitancy**	
No	259 (90.0)
Yes	40 (10.0)
**Health System Efficacy in Procuring/Distributing Vaccines**	
Yes	250 (62.7)
No	149 (37.3)

With regards to physicians’ experience with COVID-19 policies, most physicians (60%) indicated that policies implemented by their healthcare facility were adequate in handling COVID-19, only 42% specified that the policies implemented by their public health agencies were adequate (Fig 2).

### Physicians’ attitudes towards COVID-19

With regards to attitudes (Table 6), high ratings of agreement (i.e., ≥ 50% agreement) were reached regarding questions of increased workload (Item 3), subjective mental stress (Item 4), worrying about the future (Item 8), and fear of contracting the virus and passing it on to family or friends (Item 9). Importantly, most physicians (n = 247, 87%) indicated that they are willing to continue working in the health system after the pandemic (Item 10).

**Table 6:** Summary of physicians’ attitudes towards COVID-19 (N = 399).

Item	Strongly Disagree N (%)	Disagree N (%)	Neutral N (%)	Agree N (%)	Strongly Agree N (%)
**Q1:** I am afraid of working in places where patients suspected of COVID-19 infection are admitted/cared for.	83 (20.8)	124 (31.1)	91 (22.8)	79 (19.8)	22 (5.5)
**Q2:** I am afraid of treating a patient with COVID-19 infection.	100 (25.1)	129 (32.3)	69 (17.3)	75 (18.8)	26 (6.5)
**Q3:** The COVID-19 pandemic has led to an increase in my daily workload	22 (5.5)	73 (18.3)	83 (20.8)	127 (31.8)	94 (23.6)
**Q4:** Due to the COVID-19 pandemic I feel mentally strained	25 (6.3)	45 (11.3)	86 (21.6)	167 (41.9)	76 (19.0)
**Q5:** Since the outbreak of the COVID-19 pandemic, the satisfaction with my job has worsened	40 (10.0)	104 (26.1)	105 (26.3)	104 (26.1)	46 (11.5)
**Q6:** I feel left alone by the responsible political decision-makers	36 (9.0)	100 (25.1)	108 (27.1)	99 (24.8)	56 (14.0)
**Q7:** Due to the COVID-19 pandemic, I have significantly less time for my personal life	30 (7.5)	105 (26.3)	94 (23.6)	118 (29.6)	52 (13.0)
**Q8:** Due to the COVID-19 pandemic, I am worrying more often about the future	33 (8.3)	58 (14.5)	89 (22.3)	155 (38.8)	64 (16.0)
**Q9:** I fear that due to my daily exposure to COVID-19 at work I could pass it on to my friends or relatives	27 (6.8)	69 (17.3)	69 (17.3)	167 (41.9)	67 (16.8)
**Q10:** I will continue to work in the healthcare area after the COVID-19 pandemic	8 (2.0)	8 (2.0)	36 (9.0)	153 (38.3)	194 (48.6)

### Physicians’ experiences of COVID-19

**Fig 3:**
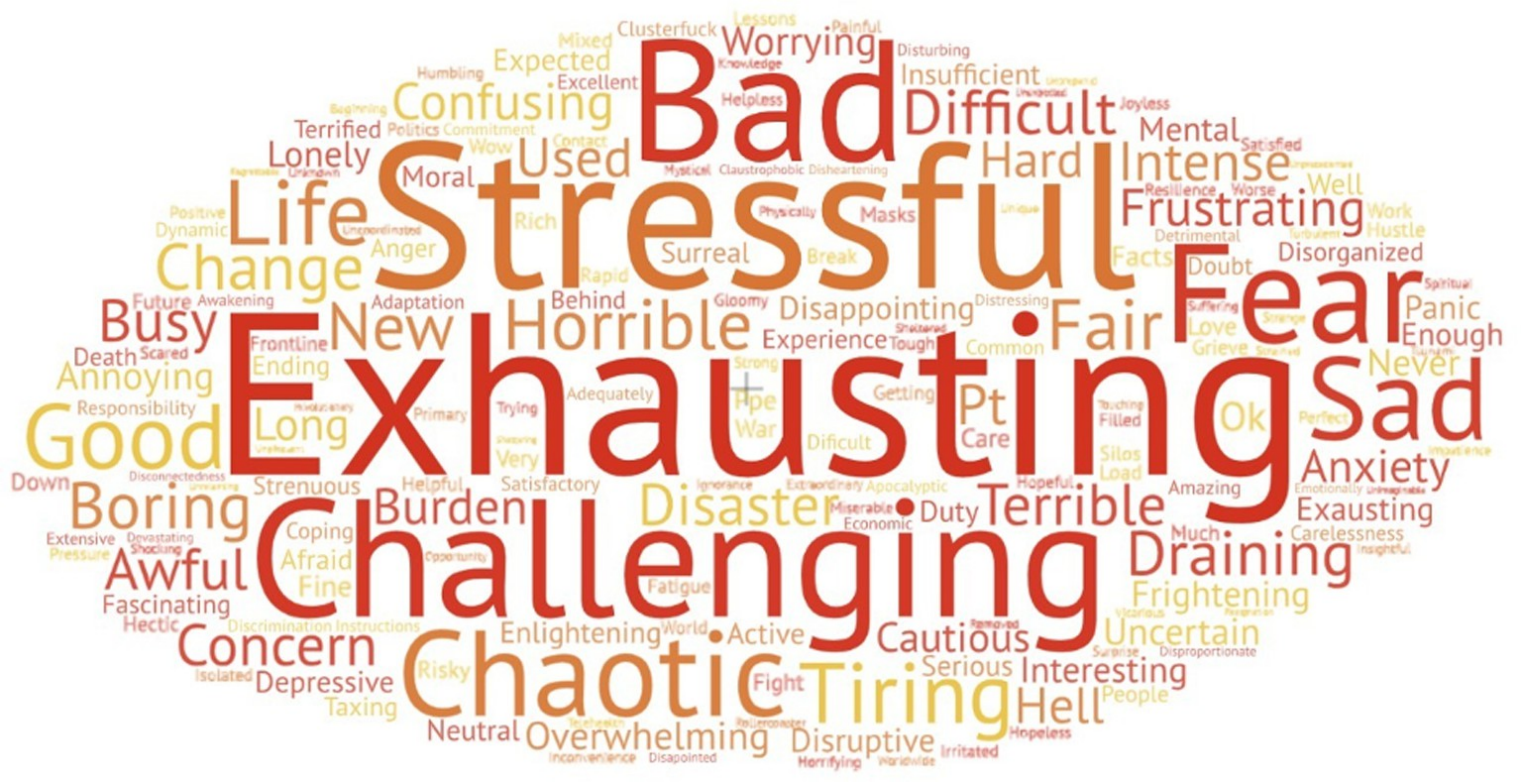
Visual representation of physicians’ experiences with COVID-19: One-word descriptions (n = 389). This word cloud depicts physician’s responses regarding their experience with COVID-19 pandemic. The bigger and bolder the word appears, the more often it was mentioned among responses. Responses were thematically analysed into three distinct themes: Negative Experiences (n = 253, 65%), Positive Experiences (n = 23, 6%), and Neutral Experience (n = 113, 29%). The top three most utilized words were “Exhausting” (n = 30), “Challenging” (n = 27), and “Stressful” (n = 22). See Table 7 for all responses.

A total of 389 participants responded to the question: Describe your experience with COVID-19 in one word. A total of 168 unique words were organised under 20 descriptive subthemes and subsequently grouped into three major themes, namely (1) Negative experience (n = 253, 65%), (2) Positive experience (n = 23, 6%), and (3) Neutral experience (n = 113, 29%). Fig 3 presents a visual representation of physicians’ experiences of COVID-19 (n = 389) one-word descriptions. Table 7 summarises the thematic analysis of physician experiences. No significant differences between demographic variables (including physician specialties, frontline worker status, or residency in LMIC versus HIC) were observed in association with a negative, positive, or neutral experience. However, participants who indicated the policies implemented by their healthcare facilities were inadequate (i.e., disorganized or inefficient) were more likely to also describe their experience with COVID-19 using negative terminology (X^2^(6) = 29, p < 0.0001). There were no differences in one-word responses based on participants’ perspectives on policies implemented by public health agencies.

**Table 7:** Summary of thematic analysis of physicians’ experiences with COVID-19 (N = 389).

Theme	Sub-theme (N)	Words
**Negative Experience**	All-Consuming (91)	Anxiety (3), Worrying (3), Draining (6), Exhausting (30), Fatigue, Overwhelming (5), Strenuous (3), Stressful (22), Taxing (2), Tiring (9), Tough, Trying, Fight for life, Long (2), Never-ending (2)
	Appalling/Agony (37)	Suffering, Awful (3), Bad (10), Burden (2), Detrimental, F****D, Hell (2), Horrible (8), Miserable, Painful, Shattering, Terrible (5), Worse
	Resentment (23)	Anger, Annoying (3), Boring (4), Disappointing (5), Disturbing, Frustrating (7), Irritated, Inconvenient
	Depressive (20)	Death, Depressive (2), Disheartening, Distressing, Gloomy future, Grieve, Helpless, Hopeless, Joyless, Mental break down, Regrettable, Sad (6), Unpleasant, Resignation
	Frantic (20)	Chaotic (9), Claustrophobic, Clusterfuck, Disruptive (3), Hectic, Panic (2), Pressure-filled, Risky, Impatience
	Fear (18)	Afraid, Fear (9), Frightening (4), Horrifying, Scared, Terrified (2)
	Uncertainty (14)	Concerning (4), Confusing (5), Doubt, Turbulent, Uncertain (3)
	Catastrophic (11)	Devastating, Apocalyptic, Tsunami, Unimaginable, Unrelenting, War, Disaster (5)
	Inadequate (11)	Behind, Discrimination, Disproportionate, Ignorance, Insufficient (2), Uncoordinated, Unprepared, Carelessness, Disorganized (2)
	Seclusion (8)	Disconnected, Isolated, Lonely (2), No contact, Removed, In silos, Sheltered
**Positive Experience**	Worthwhile (11)	Excellent, Good (6), Love frontline work, Perfect, Positive
	Illuminating/Revealing (10)	Amazing, Hopeful, Awakening, Enlightening (3), Extraordinary, Fascinating (2), Insightful
	Beneficial (2)	Helpful, Useful
**Neutral Experience**	Demanding (35)	Difficult (6), Hard (2), Challenging (27)
	Unparalleled (23)	Spiritual, Interesting (4), Life-changing (2), Revolutionary, Strange, Surreal (2), Unique, Unknown, Unprecedented, Vicarious, World-changing, Wow, Humbling, Surprise, Unexpected, Shocking, Touching, Mystical
	Adequate (14)	Fair (2), Fine, Not bad, Okay, Satisfactory (3), Mixed, Neutral (2), Expected (2), Life
	Significant/Substantial (13)	Rich, Serious (2), Extensive, Intense (4), Strong, Cautious (2), Only the beginning, Worldwide
	Physicians’ Duty (11)	Commitment, Opportunity, Primary care experience, Responsibility (2), Telehealth, Work Load, Frontline, Experience, PPE, Politics
	Adjustment (10)	Adaptation, Change (2), Getting used to it, Lessons, Resilience, Coping well, New (3)
	Immersive (7)	Active, Busy (2), Dynamic, Hustle, Rollercoaster, Rapid

### Physicians’ recommendations for future pandemics

A total of 387 participants responded to the question: What recommendations do you have for future pandemics? Inductive thematic analysis of responses revealed twenty-seven distinct subthemes organised into seven major themes, described below. Table 8 summarises physicians’ recommendations, and S1 Table provides detailed codes and exemplar quotes from the thematic analysis.

**Table 8:** Summary of thematic analysis of physicians’ recommendations (N = 387).

Theme	Summary
Holistic Preparation	Physicians acknowledged the importance of preparing for future pandemics through education, prevention, proactive planning, and pre-emptive policy development and implementation.
Execution of Response Measures	Physicians expressed the need of recognizing pandemics, implementing better guidelines, minimizing response time, adequately implementing response measures, in addition to improvements in surveillance and vaccination.
Health System Strengthening	Physicians recognized the cracks in our current healthcare system and recommended strengthening the infrastructure, promoting transparency, and one that does not operate for profit.
Appropriate Delegation of Roles	Physicians observed a need to allow individuals to perform within their designated roles when managing pandemics.
Minimize Infodemics	Physicians indicated the importance of minimizing the spread of misinformation during pandemics by improving communication in addition to ensuring the distribution of credible information.
Global Responsibility	Physicians acknowledged the importance for global unity when managing global outbreaks and establishing pandemic-resistant global health systems.
Uncertainty	A few physicians were uncertain about providing recommendations or expressed limitations of their role as a physician in being able to provide recommendations.

#### Theme 1: Holistic preparation

This theme represents preparing for future pandemics through education, prevention, and pre-emptive policy development and implementation. Physicians called for “more research” on pandemics and ensuring that the public, politicians, and interdisciplinary medical teams were continuously educated on the risks of a pandemic. One physician recommended to “build specific structures against pandemics”, while another suggested to “minimize human-animal interactions”. Mostly however, physicians specified to “learn from mistakes” and “ensure that any knowledge gained from the past is applied proactively for future pandemics”. One physician voiced the need to “have a better pandemic preparedness strategy, don’t wait for the 2nd/3rd wave” and another revealed “now is the time to prepare”.

#### Theme 2: Execution of response measures

This theme included actionable items relating to pandemic response and highlighted the importance of attending to the emotional wellbeing of people. On the threat of the pandemic, physicians pointed out the need for “less denial” and to “take it seriously earlier”. Others revealed the need for accountability: “the country responsible for the outbreak must take responsibility and admit”. Some physicians (n = 9, 2%) called for clear and standardised guidelines, “have a manual of operation and follow it”. However, a need for flexibility was also voiced, “allow MDs to treat patients according to their judgement and do not limit them to strict guidelines”. Additionally, physicians proclaimed a need to “act quickly and definitively” and “be rapid and safe in your response”. Some physicians noted the need for “early diagnosing, tracing, and isolating cases”. Others recommended implementing stricter protective measures stating: “quarantines should be stronger”, “earlier ban in travel”, and “mask mandates”. Physicians expressed the need for local decision making, “decision-making at the local and state levels according to the degree of incidence”. A portion of physicians agreed on the significance of vaccinations, recommending better accessibility, compliance, and distribution of vaccinations. They noted a need for the world to “achieve herd immunity through vaccination” and some called for “mandatory vaccination”. Physicians also recommended addressing the morale of the public: “don’t panic”, “be realistic”.

#### Theme 3: Health system strengthening

Physicians recommended for health systems with stronger infrastructure and comprehensive resources for its physicians. Physicians also called for a health system that prioritises “physician health and safety” and promotes transparency among its constituents. Some physicians voiced a need for the health system to employ an interdisciplinary approach: “We should have programs that cover the entire spectrum from physical, psychological, social, and spiritual health as a continuum”. Another physician highlighted the “need to develop medical infrastructure in low-income countries”. Physicians suggested having a system that allows for “decentralized participatory planning on part of government agencies”. Physicians also communicated the need for a health system that is “not business oriented” and “invests more in mental health and financial support of entire population”. To strengthen the health system, physicians expressed a demand for adequate material resources (e.g., “have adequate stock of PPE”), additional human resources (e.g., “provide more trained manpower”), “improve epidemic control centres”, and an “established task force all year round”.

#### Theme 4: Appropriate delegation of roles

Physicians specifically highlighted the role of politicians and their responsibility to form a more “empathetic political system” that can respond to the pandemic. Physicians (n = 34, 7%) stated a need to differentiate the role of science and healthcare professionals from the role of politicians. This was recommended particularly during policy-development, “strengthen the position of the clinicians in the decision-making”. On several occasions, physicians recommended the need for “less politics, more science”, that “policymakers should listen more to health professionals’’, and that “politicians should stop managing what they can barely comprehend”. Additionally, physicians recommended, “put public health physicians and epidemiologists at the front”. Other physicians focused on the role of the WHO and the need to “re-organize it”.

#### Theme 5: Minimise adverse effects of infodemics

This theme captures physicians’ input towards minimising the spread of misinformation. Physicians particularly called for “much better and more timely public health communications needed” and to “improve social communication to avoid fake news”. Physicians stated a need to ensure that the content of information distributed is relevant and credible, “Prevent fake news from spreading, if possible. People believe it.” With regards to inter-departmental communication, physicians recommended standardising and/or centralising the distribution of pandemic-related communications, “One central body and not 50 different emails about the same advice from different departments”. On the role of communication with the public, “Don’t let social media give information to the public without peer review. The information system must be more open (data access) but it is necessary to identify the right communicator.”

#### Theme 6: Global responsibility

This theme encompasses physicians’ views on the significance of global unity during pandemics. Physicians recommended global action through “better planning with pandemic resistant health systems”. Physicians also indicated the necessity for global transparency, one wrote: “China did respond too slowly and did not communicate about the severity of the situation and did not react to control outbreak”. Physicians also emphasised a need for “global coordination, solidarity, and equity”, and stated that “the world needs to learn to work together”. Additionally, physicians specified demands for a “global initiative to reduce social inequality” and “equitable vaccine distribution all over the world”.

#### Uncertainty

Few physicians expressed uncertainty towards providing recommendations, stating they were “unsure”. Other participants acknowledged the limitations of their role in being able to provide recommendations, one respondent explicitly noted “I’m not a public health expert!”.

## Discussion

### Main findings in light of other evidence

The results of this global survey revealed international agreement on the burden of care experienced by physicians during the COVID-19 pandemic and particularly when working in underprepared communities or institutions. Most physicians in this study possessed good overall knowledge of COVID-19; this is in line with previous studies [36–38]. Additionally, physicians relied on official government websites as their primary source of information, as supported by an earlier study among HCPs [24]. This suggests that physicians have been consistently utilising reliable sources to acquire information regarding COVID-19 and correlates with the good knowledge observed. However, respondents in this study exhibited poor knowledge on domains relating to the nature and treatment of disease. Previous studies on this are inconsistent, with some physicians displaying good knowledge of the disease [39] and others showing poor knowledge [40]. The discrepancy between studies could be due to differences in programmes delivered by health facilities in supporting and educating physicians, reduced accessibility to evidence-based information in some settings, as well as differences in national-level protocols for the management and treatment of disease. With regards to preventative practices towards COVID-19, many physicians reported occasionally wearing masks and/or gloves. Although this may reflect poor adherence to safety measures by physicians, it could also be due lack of available or accessible Personal Protective Equipment (PPE) such as masks and/or gloves. Access to PPE was particularly limited in both HICs and LMICs during the initial stages of the pandemic due to lack of preparedness of health systems, disruption of global supply chains and mismanagement [41, 42].

Almost all physicians in our study indicated that they have received the COVID-19 vaccine, and only a small percentage were/are hesitant to receive the vaccine. The degree of vaccine hesitancy among this population of physicians is echoed in other studies [43]. Additionally, about half of physicians in this study were concerned about the rapid development of vaccines. Data from HICs suggests the rapid pace of vaccine development as one of the primary reasons for vaccine hesitancy [44].

COVID-19 revealed a lack of adequate policies, preparedness, and education necessary to combat a pandemic and control further outbreaks [45–47]. Further, the implementation of rapid pandemic control measures was at times delayed [48]. Our survey results indicate that many physicians perceived the policies and actions implemented by their healthcare facilities and public health agencies as being insufficient, which correlated with physicians’ overall experience with the COVID-19 Pandemic, where those who perceived their facilities as having inadequate policies were more likely to also describe their experience using negative terminology. Additionally, many physicians recommended a need to strengthen healthcare and political systems to better respond to pandemics. These findings are in line with physicians’ demands for better resources for future pandemics, since a better equipped health and political system is more likely to provide the necessary resources to tackle the pandemic. Previous studies support such recommendations, especially for evidence-based policy-making as a means to bridge the gap between clinical science and policy during the pandemic [49–52]. It is also recognised that policies to combat infectious disease outbreaks must be implemented rapidly while also meeting the needs of multiple sectors including public health, economy, and social welfare [53]. Implementing a One Health approach, recommended in this study, is crucial as the efforts of one sector, or many sectors working in silos, cannot eliminate the threat of a pandemic. As suggested by physicians in this study, the WHO has a unique responsibility in helping countries, especially LMICs, prepare for pandemics, as well as supporting efforts to initiate and mount an effective response. These recommendations highlighted the significance of early detection, risk communication with vulnerable groups, strategies for containment, and international collaboration [54].

The spread of misinformation during previous pandemics led to confusion, risk-taking behaviours, and mistrust between the public and healthcare professionals [55–57]. Furthermore, within a highly digital society, the risks of ‘infodemics’ could be dependent on effective communication strategies that counter unreliable news [58]. Hence, the recommendations in this study for better communication strategies are much warranted.

The call for global unity during pandemics, echoed by physicians in this study, is also essential. According to the Global Dashboard for Vaccine Equity, as of May 18, 2022 only 17.61% of individuals in low-income countries have been vaccinated with at least one dose, in comparison to 72.23% in high-income countries [59]. The continued inequitable vaccine distribution leaves millions of individuals vulnerable to being infected by COVID-19 and promotes the emergence and subsequent spread of deadly variants across the globe.

### Recommendations for future policy development

In our study, physicians provided recommendations regarding future interventions and/or policies that may help mitigate the impact of future pandemics on civilians and healthcare professionals. Physicians recommended a strong need to:

1. Strengthen health systems by preparing the healthcare sector for future pandemics; suggestions included to (a) invest in virology research, (b) train HCPs, (c) develop guidelines pre-emptively, and (d) arrange an emergency stockpile of material resources for clinicians including PPEs.
2. Prevent infodemics by having healthcare professionals collaborate with politicians and social media outlets to guarantee that credible information is being sourced to the public.
3. Delegate decision-making roles appropriately, by promoting an empathetic political system that understands the need for input from scientists and HCPs in dictating best-practices for pandemic management.
4. Acknowledge global responsibility and the necessity for international collaboration and equity. This must be done by collaborative preparation and prevention as well as through the equitable distribution of resources.

### Strengths and limitations

This is the first up to date mixed-method global study, to our knowledge, with a large sample size (399 physicians) in 62 unique countries including high and low- and middle-income settings. The survey questionnaire was also developed based on the most recent information from the WHO and was subsequently validated and piloted prior to distribution. Additionally, this study included both quantitative and qualitative findings, ensuring that the results obtained are grounded in participants’ experiences and allowing for better translation and implementation of population and behavioural research [60, 61].

We acknowledge the following limitations. Online surveys pose specific challenges including the inability to calculate response rate, the potential for the data to not be representative, and the possibility of recall bias. A further limitation of social media research is inability to ensure respondents are truly physicians. To mitigate this limitation, our team (a) contacted known physicians directly, (b) included a screening question in the survey asking about physician status, and (c) collected data from more than the minimum sample size required for reliability. Snowballing may have also introduced bias, as participants identified in that way may share similar opinions [31]. Moreover, the survey does not account for local differences in pandemic response and management. Additionally, the results may not reflect the new knowledge acquired after the study, in particular the emergence of new variants and the introduction of new guidelines and practices. However, as new variants continue to emerge, the recommendations that physicians have expressed, in particular strengthening health systems and global collaboration, should be taken into consideration when developing guidelines. Lastly, the survey was designed and written in English, potentially introducing response bias.

## Conclusion

Findings from this global survey indicated that most physicians possessed good knowledge of COVID-19 disease yet limited adherence to safety measures. Physicians were particularly concerned about the distribution of vaccines to the general population, and approximately one third indicated that the policies implemented by their healthcare facilities and public health agencies were insufficient in handling the pandemic. Although most physicians described their experience with COVID-19 in negative emotive language and agreed that the pandemic had led to increased mental stress, most were willing to continue working in the healthcare sector post-pandemic. Collectively, this study suggests that physicians may need to have a more dominant role in policymaking in addition to their role as clinical experts. Given that future pandemics are inevitable [62], exploring how and in what capacity clinicians will contribute to policy-making processes during health emergencies could be crucial.

## Supporting information

S1 Table List of categories, themes, codes, based on thematic analysis of physicians’ recommendations for future pandemics. (DOCX)

S1 File Study questionnaire. (DOCX)
