# Supplementary material for: Knowledge and experience of physicians during the COVID-19 Pandemic: A global cross-sectional study
Source: PLOS Glob Public Health. 2022 Jul 29;2(7):e0000639. doi: 10.1371/journal.pgph.0000639 (PMC10022385; doi:10.1371/journal.pgph.0000639)
Supplement: S1 Table — (DOCX) [file pgph.0000639.s001.docx]

## Supplementary Table 1. List of categories, themes, codes, based on thematic analysis of physicians’ recommendations for future pandemics.

| **Analytical Theme** | **Descriptive Theme** | **Code (N=529)** | **Representative Quotes** |
| --- | --- | --- | --- |
| Uncertainty (6) | | | • I'm not a public health expert!  • Difficult to predict future  • No idea |
| Holistic Preparation | Education (30) | Education (6)  Education - Politicians (1)  Education - Public (5)  Education - Medical, Interdisciplinary (1)  HCP Training (3)  HCP Training - in ICU (1)  HCP Training- For Unpredictable Situations (1)  Research (10)  Research - Funding (1)  Research - Training (1) | • Public health education on a continuous basis is fundamental  • Public must be constantly educated  • Invest in education as early and often as possible  • Educating politicians  • Train medical students and other health professions to deal with such unpredictable situations  • More research on the pandemic |
|  | Prevention (24) | Prevention (19)  Prevention - Prevention > Treatment (1)  Vegetarianism (2)  Zoonosis - Minimize (2) | • Prevention to build specific structures against pandemics en epidemics infections  • Minimize human-animal interactions |
|  | Proactive Planning (82) | Precaution (2)  Learn From Experiences (7)  Learn From Mistakes (6)  Anticipation (2)  Planning (11)  Planning - Early (2)  Planning - Innovated (1)  Preparation (41)  Preparation - Proactive (1)  Preparation - Alert (3)  Preparation - Emergency Planning (3)  Preparation - Financial (2)  Preparation - Resources (1) | • Maintain the expertise gained by this one  • Ensure that any knowledge we gained from the past is applied proactively for future pandemics  • Spend more time and resources in the phase of preparedness  • Have a better pandemic preparedness strategy, don't wait for 2nd/3rd wave  • Now is the time to prepare  • Be prepared/expect/learn lessons from this |
|  | Policy Development and Implementation (13) | Policies (2)  Preparation - Policies (2)  Policies - Comprehensive, Integrated (1)  Policies - Early (2)  Policies - Evidence Based (1)  Policy - Precaution and Prevention for Future (1)  Policy - Update (2)  Policy - Masks (1)  Policies - Mandate It (1) | • Preparedness and protocols for rapid response  • Good, comprehensive, consistent, and integrated policy from the government since the start  • Normalize shift in policies with new evidence |
| Execution of Response Measures | Initial Recognition (8) | Acceptance (1)  Acceptance - Early (1)  Awareness (4)  Accountability (1)  Accountability - Punishment (1) | • The country responsible for the breakout must take responsibility and admit  • Take it seriously earlier  • Punish the countries that spread such viruses |
|  | Guideline Implementation (9) | Guidelines (2)  Guidelines - Follow (1)  Guidelines - Leniency, Management (Drugs) (1)  Guidelines - Social Distancing (1)  Guidelines - Standardized Protocols (1)  Sanitation (2)  Sanitation - Wash Hands (1) | • Have a manual of operation and follow it  • Allow MDs to treat patients according to their judgement and do not limit them to strict guidelines  • Standard protocol of management  • Clear guidelines |
|  | Response Time (27) | Response - Early Management (4)  Response - Proactive (3)  Response - Proactive > Reactive (1)  Response - Quick Government (1)  Response - Quick/Early (15)  Response - Rapid, Safe (3) | • Act quickly and definitively  • Implement lockdowns and other measures sooner  • Quick reaction from government  • Be rapid and safe in your response |
|  | Response Measures (30) | Response - Active, Lockdowns (1)  Response - Containment (2)  Response - Isolation (3)  Response - Lockdown (3)  Response - Protective Measures (2)  Response - Stronger Quarantine (1)  Response - Travel Restrictions (2)  Response - Mask Mandates (2)  Prioritize Vulnerable (1)  Organization (9)  Organization - Hospital Wards (2)  Organization - Workplace Efficiency (1)  Telehealth (1) | • As early as possible closure of the place of origin of infection  • Early diagnosing, tracing, and isolating cases  • Focus on most vulnerable  • Quarantine should be stronger  • Work efficiency and better organization  • Hospital should reorganize wards and segregate covid positive versus negative to minimize spread of infection |
|  | Response Development (8) | Response - Coordinated (2)  Response - Local Level Decision Making (1)  Response - Holistic/Balanced (1)  Response - Physician Directed (1)  Response - Standardized (2)  Response - Innovated (1) | • Allowing physicians to direct it, not government bureaucrats, non-physician practitioners, or politicians  • Decision-making at the local and state levels according to the degree of incidence |
|  | Surveillance (21) | Response - Identification/Detection (2)  Response - Investigations (1)  Response - Surveillance - Contact Tracing (2)  Response - Primary Control (1)  Response - Screening (1)  Control (2)  Control Spread (1)  Surveillance (7)  Surveillance - Contact Tracing, Testing (2)  Surveillance - Containment (2) | • Early detection  • Close borders as soon as possible or do more public effort in screening  • Surveillance for cases and early institution of control of spread measures  • Effective surveillance for rapid response |
|  | Vaccination (44) | Vaccination (21)  Response - Vaccination (1)  Vaccination - Accessibility (2)  Vaccination - Compliance (2)  Vaccination - Herd Immunity (1)  Vaccination - Early (1)  Vaccination - Education (1)  Vaccination - Efficacy, Quantity (1)  Vaccination - Elderly (1)  Vaccination - Mandate It (4)  Vaccination - Manufacturing (2)  Vaccination - Distribution (7) | • More access to vaccines   There is legal precedent for mandatory vaccination  • World achieves herd immunity through vaccination with good distribution  • Capacity for local vaccine production  • We need effective vaccines in adequate quantity |
|  | Emotional Wellbeing Response (16) | Adaptation (4)  Morale (3)  Morale - Calm (2)  Morale - Acceptance (1)  Morale - Panic (1)  Morale - Realistic (1)  Morale - Survival (1)  Reflection (3) | • Resumption of activities of daily living in the city in a safe way  • Be realistic  • Don't panic  • Survival Mode  • Reflection on our way of life |
| Health System Strengthening | Health System Infrastructure (15) | HS - Advocacy for Patients (1)  HS - Adaptation (1)  HS - Early Symptom Recognition, Communication and Treatment (1)  HS - Funding (1)  HS - Infrastructure, Public Welfare, Public Mental Health Programs (2)  HS - Leadership/Workforce (1)  HS - Infrastructure - Decentralized Planning (1)  HS - LMIC Infrastructure (1)  HS - One Health Approach (2)  HS - Strengthening MOH (1)  HS - Transform Care Models (1)  HS Strengthening (1)  HS Strengthening - Prioritize Healthcare Practitioners (1) | • Decentralized participatory planning on part of government agencies  • The One Health Approach. For patients and physicians we should have programs that cover the entire spectrum from physical, psychological, social and spiritual health as a continuum.  • Need to develop medical infrastructure in low income countries  • Increase resources in public health and onehealth  • Healthcare needs to change and adapt |
|  | Physician Health (4) | Physician Health/Safety (2)  Physician Mental Health (2) | • Take care of your doctors  • Check on your mental health |
|  | Interdisciplinary Collaboration (19) | Interdisciplinary Collaboration (11)  Coordination (1)  Coordination - Government Politics (6)  Collaboration (1) | • More coordinated and consistent responses between health care leaders and government leaders  • Holistic approach to population wellbeing needs to be considered |
|  | Not for Profit (5) | Not for Profit (1)  Not for Profit - Human Misery (1)  Not for Profit - Not Business Oriented (1)  Not for Profit - Pharma Companies (1)  Not for Profit - Free for All (1) | • Force pharmaceutical companies to stop making profit  • Don't profit from human misery and don't be predatory  • Science-based leadership above profits |
|  | Resources (27) | Resources (2)  Resources - Free Testing (1)  Resources - Hand Hygiene (1)  Resources - HS Infrastructure (3)  Resources - Human Resources (5)  Resources - Masks (3)  Resources - PPE Accessibility (1)  Resources - PPE (3)  Resources - Public Health (1)  Resources - Share Supplies (1)  Resources - Surveillance, Testing (1)  Resources - Screening (1)  Resources - Task Force (3)  Resources - Prevention (1) | • Share best practices; supplies  • Have adequate stock of PPE  • Ensure PPE for all healthcare system workers  • Creation of expert committees  • More resources  • Increase resources in public health  • Country preparedness from a human resources for health and financial perspective  • Improve epidemic control centers  • Implement an established task force all year round |
|  | Transparency (15) | Transparency (12)  Transparency - Biopharma (1)  Transparency - Leadership (1)  Transparency - Trust (1) | • Acknowledgment from all leadership unfront that we are learning as we go and transparency is key  • Collective collaboration with transparency  • More transparency |
| Appropriate Delegation of Roles | Political Responsibility (3) | Politics - Change Government (1)  Politics - Empathy (2) | • A more empathetic political system  • Change the government |
|  | WHO (1) | Role of WHO (1) | WHO is a disgraceful organization, reorganize it. |
|  | Science vs. Politics (34) | Frontline Opinion (1)  Science (5)  Science vs. Politics - Agencies (2)  Science vs. Politics - Keep Them (“Politics”) Out (12)  Science vs. Politics - Epidemiologists (1)  Science vs. Politics - Opinions (11)  Science vs. Politics - Policy Making (2) | • Strengthening position of clinicians in decision-making, political framing  • Less politics, more science  • Policymakers should listen more to health professionals  • Pay attention to epidemiologists' recommendations |
| Minimize Infodemics | Communication (26) | Communication (4)  Communication - Public Awareness (3)  Communication - Centralized (1)  Communication - public motivation (1)  Communication - Science Group (1)  Communication - Social Media Misinformation (5)  Communication - Avoid Misinformation (1)  Communication - Timely (4)  Communication - with Public (3)  Communication - Inclusive (1)  Communication - Spokesperson (1)  Communication - Evidence-Based (1) | • Much better and more timely public health communications needed  • Improve social communication in order to avoid fake news  • Don't let social media to give the information to the public without peer review. The information system must be more open (data access) but is necessary to identify the right communicator  • Prevent fake news from spreading, if possible  • Better understanding of people's motivation in public health |
|  | Information (21) | Information (3)  Information - Censorship (1)  Information - Credibility (4)  Information - Media Coverage (2)  Information - Relevance (1)  Information - Standardized (3)  Information - Transparency (2)  Information - Availability (1)  Information - Sharing (1)  Information - Infodemic (1)  Misinformation (1)  Misinformation - Public (1) | • Availability of true information for action  • Consistency of advice from day to day  • Less infodemic  • Do away with misinformation  • Share relevant information  • Allow and encourage scientific debates instead of censorship  • One central body and not 50 different emails about the same advice from different departments |
| Global Responsibility | Global Equity (9) | Global Equity (9) | • Global initiative to reduce social inequality |
|  | Collaboration (23) | International Collaboration (19)  International Collaboration - Information Sharing (3)  International Collaboration - Preparation, Resources (1) | • Better world connection  • The world needs to learn to work together |
|  | Global Action (9) | Planning - Global Health System (2)  Response - Standardized Therapy Globally (1)  Vaccination - Global (5)  Transparency - Global (1) | • Equitable vaccine distribution all over the world  • International transparency, China did respond to slowly and did not communicate about severity of situation and did not react to control outbreak |
