# Supplementary material for: Knowledge and experience of physicians during the COVID-19 Pandemic: A global cross-sectional study
Source: PLOS Glob Public Health. 2022 Jul 29;2(7):e0000639. doi: 10.1371/journal.pgph.0000639 (PMC10022385; doi:10.1371/journal.pgph.0000639)
Supplement: S1 File — (DOCX) [file pgph.0000639.s002.docx]

## Supplementary Text 1. Study questionnaire

Study Title: *Knowledge and Perspectives of Health Care Providers on COVID-19: A Global Cross-sectional Study*

REC Reference Number: 2021.0127

*I am an MBBS* Medical Student at the *St George’s University of London*. I would like to invite you to participate in this anonymous survey which forms part of my research activities. You should only participate if you want to; choosing not to take part will not disadvantage you in any way. This study has been given a favourable ethical opinion by the St George`s Research Ethics Committee.

**What is the research about?**

The aim of this study is to investigate knowledge and perspectives of physicians on COVID-19 and its various strains. I am specifically interested in understanding your experiences working throughout the COVID-19 pandemic, your sources of knowledge, your perspectives on health policies implemented as well as your perspective on the various vaccinations available today.

I am inviting doctors to participate in this survey.

**What will happen to me if I take part?**

If you chose to take part, you will be asked to fill in this questionnaire, which is completely anonymous. The demographic data will be collected as gender, age, profession, speciality, and country of residence.

The questionnaire will take 5-7 minutes to complete.

The submission of a questionnaire is implying consent.

The incomplete datasets will not be included in the analysis.

Research data will be stored on a secure, password-protected drive (Microsoft OneDrive) and will be retained for 5 years.

**Can I change my mind?**

Participation is completely voluntary, and you can stop at any time. Please note that anonymous data cannot be withdrawn after they have been submitted.

**What are the possible benefits and risks of taking part?**

The information we will get from the study will inform future interventions and policies aimed at educating and supporting healthcare providers during the current and future pandemics.

We do not expect you to find the questions upsetting, but in the unlikely case that you do, please contact our team and we will put you in touch with the nearest counselling service.

**Where can I get more information?**

[m2007027@sgul.ac.uk](mailto:m2007027@sgul.ac.uk)

**The online survey runs from 06 August to 23 August 2021.**

**Thank you for reading this information section and for considering taking part in this research.**

**Check this box to indicate you have read and understood this information section, and that you consent to participate in this survey**

**Questions:**

**Section 1: Sociodemographic Info**

- Gender
  - Female
  - Male
  - Transgender
  - Non-Binary
  - Other
- Age (years)
  - Younger Than 25
  - 25 - 35
  - 36 - 45
  - 46 - 55
  - 56 - 65
  - 66 - 75
  - Older Than 75
- Profession
  - Physician
  - Nurse
- Specialty
  - General Practitioner
  - Internal Medicine
  - Paediatrics
  - Emergency Medicine
  - Obstetrics Gynaecology
  - Surgery
  - Other _________
  - N/A
- Place of Work
  - Private Establishment
  - Public Establishment
- Frontline Worker
  - Yes
  - No
- Years of Experience
  - < 10 years
  - ≥ 10 years
- Country of Residence (Drop Down Menu with List of Countries)

**Section 2: Sources of Information**

On a scale of 1 "least used sources" to 4 "most used sources", how do you rank your sources of information about the COVID-19 pandemic?

|  | Least used | Sometimes | More often | Most used |
| --- | --- | --- | --- | --- |
| News, Media (TV, Radio, newspapers etc.., (1) |  |  |  |  |
| Social media (Facebook, Twitter, Whatsapp, YouTube, Instagram, Snapchat…) (2) |  |  |  |  |
| Official government websites (MOH, DHA, DOH, WHO, CDC…..) (3) |  |  |  |  |
| Family member, colleague or friend (4) |  |  |  |  |

**Section 3: COVID Knowledge**

**Nature of the Disease**

K1: What is the incubation period of COVID-19?

1. 2-7 days
2. 2-14 days
3. 7-14 days
4. 7-21 days
5. None of the above

K2: COVID-19 origin is thought to be from:

1. Bats
2. Snakes
3. Fish
4. Camel
5. Unknown

K3: The COVID-19 variants have different clinical manifestations.

1. True
2. False

K4: What are the complications of COVID-19?

1. Pneumonia
2. Respiratory failure
3. Death
4. Thromboembolism
5. Sepsis
6. All the above

**Transmission of the Disease**

K5: COVID-19 transmission occurs through:

1. Air
2. Contact
3. Faecal-Oral
4. All the above
5. None of the above

K6: The UK and Indian variants of COVID-19 spread faster as they are more transmissible or infectious.

1. True
2. False

**Actions in Dealing with Suspected, Probable and Confirmed Cases**

K7: The use of personal protective equipment is necessary during aerosol production procedures, such as suction sputum sampling and intubation.

1. Yes
2. No
3. Don’t Know

K8: Suspected cases of COVID-19 infection after triage should be taken into care in a negative pressure respiratory isolation room.

1. Yes
2. No
3. Don’t Know

K9: The use of N95 masks is necessary when sampling of induced sputum from patients suspected of COVID-19 infection.

1. Yes
2. No
3. Don’t Know

**Treatment of Disease**

K10: Oxygen therapy should be given to all cases of severe COVID-19 with acute respiratory infection.

1. Yes
2. No
3. Don’t Know

K11: High doses of systemic corticosteroids should be avoided in patients with confirmed or suspected COVID-19 infection and clinical manifestations of viral pneumonia.

1. Yes
2. No
3. Don’t Know

K12: What is the treatment for COVID-19?

1. Supportive care
2. Antiviral therapy
3. Vaccination
4. None of the above

**Nature of Vaccines**

K13: Which of the following is not a common side effect of COVID-19 vaccines:

1. Muscle Pain
2. Fever
3. Fatigue
4. Anaphylactic Reaction
5. Headache

K14: Individuals who are immunodeficient and/or pregnant can receive the COVID-19 vaccine.

1. True
2. False

K15: Children below the age of 16 can receive the COVID-19 vaccine.

1. True
2. False

K16: The COVID-19 vaccines that are currently in development or have been approved are expected to provide at least some protection against new virus variants.

1. True
2. False

**Section 4: COVID-19 Practices**

Answer the following questions on preventative practices toward COVID-19 with never, always, occasionally.

|  | **Never** | **Occasionally** | **Always** |
| --- | --- | --- | --- |
| P1: I wear a mask while performing my job |  |  |  |
| P2: I wear gloves while performing my job |  |  |  |
| P3: I wash my hands with soap or rub my hands with hydro-alcoholic gel during my work shift |  |  |  |
| P4: I put my PPE on in the following order: 1- gown, 2- mask, 3- gloves. |  |  |  |
| P5: I remove my PPE in the following order: 1- gloves, 2- do hand hygiene, 3- gown, 4- mask |  |  |  |

**Section 5: Perspectives – Vaccinations**

V1: Are you concerned about the following aspects of the COVID-19 vaccinations: (options: Yes or No)

1. Rapid Development
2. Short Term Side Effects
3. Long Term Side Effects
4. Distribution to General Population
5. Effectiveness

V2: Which of the following vaccines do you think has the highest efficacy?

1. Pfizer
2. AstraZeneca
3. Moderna
4. Janssen (Johnson and Johnson Vaccine)
5. Sputnik V
6. Sinopharm
7. Sinovac Biotech
8. Other _______

V3: Which of the following vaccines do you think has the highest potential for complications?

1. Pfizer
2. AstraZeneca
3. Moderna
4. Janssen (Johnson and Johnson Vaccine)
5. Sputnik V
6. Sinopharm
7. Sinovac Biotech
8. Other _______

V4: Have you received the COVID-19 vaccine.

1. Yes
2. No

V5: Are/were you hesitant to receive the vaccine?

1. Yes
2. No

V6: Do you believe your health system has effectively procured and distributed the COVID-19 vaccinations?

1. Yes
2. No

**Section 6: Perspectives – Health System**

HS1: What are your perceptions towards policies/actions implemented by your public health agency in fighting COVID-19?

1. Acceptable/appropriate
2. Insufficient
3. Disarray/disorganized
4. Excessive and unnecessary

HS2: What are your perceptions towards policies/actions implemented by your health care facility in fighting COVID-19 (e.g., provides adequate PPE)?

1. Acceptable/appropriate
2. Insufficient
3. Disarray/disorganized
4. Excessive and unnecessary

**Section 7: Attitudes – Subjective Burden**

Answer the following questions about subjective attitudes towards COVID-19 with agree, neutral, or disagree.

|  | **Agree** | **Neutral** | **Disagree** |
| --- | --- | --- | --- |
| SB1: I am afraid of working in places where patients suspected of COVID-19 infection are admitted/cared for. |  |  |  |
| SB2: I am afraid of treating a patient with COVID-19 infection. |  |  |  |
| SB3: The COVID-19 pandemic has led to an increase in my daily workload |  |  |  |
| SB4: Due to the COVID-19 pandemic I feel mentally strained |  |  |  |
| SB5: Since the outbreak of the COVID-19 pandemic, the satisfaction with my job has worsened |  |  |  |
| SB6: I feel left alone by the responsible political decision-makers |  |  |  |
| SB7: Due to the COVID-19 pandemic, I have significantly less time for my personal life |  |  |  |
| SB8: Due to the COVID-19 pandemic, I am worrying more often about the future |  |  |  |
| SB9: I fear that due to my daily exposure with it at work I could pass on the coronavirus to my friends or relatives |  |  |  |
| SB10: I will continue to work in the healthcare area after the COVID-19 pandemic |  |  |  |

**Section 8: Personal Reflections** (open-ended questions)

PR 1: Describe your experience with the COVID-19 pandemic in one word.

PR 2: What recommendations do you have for the management of future pandemics?
